# Bridging Distances and Enhancing Care: A Comprehensive Review of Telemedicine in Surgery

**DOI:** 10.7759/cureus.76099

**Published:** 2024-12-20

**Authors:** Andrew Wanees, Ranj Bhakar, Rezuana Tamanna, Nur Jenny, Momen Abdelglil, Mohamed A Ali, Gowri M Pillai, Amina amin, Jeeva K Sundarraj, Hany Abdelmasih, Reda H Mithany

**Affiliations:** 1 General Surgery, Ain Shams University Hospitals, Cairo, EGY; 2 Trauma and Orthopedics, Torbay Hospital, Torbay, GBR; 3 General Surgery, Watford General Hospital, Watford, GBR; 4 General Surgery, Royal Victoria Infirmary, Newcastle upon Tyne, GBR; 5 Pediatric Surgery, Mansoura University Children Hospital, Mansoura, EGY; 6 Surgical Oncology, National Cancer Institute, Cairo, EGY; 7 Surgery, Bronglais Hospital, Wales, GBR; 8 General Surgery, James Cook University Hospital, Middlesbrough, GBR; 9 Vascular Surgery, Birmingham Heartlands Hospital, Birmingham, GBR; 10 General Surgery, University Hospital Wishaw, Glasgow, GBR; 11 Acute Medicine, Stoke Mandeville Hospital, Aylesbury, GBR; 12 Colorectal Surgery, Torbay and South Devon NHS Foundation Trust, Torbay, GBR

**Keywords:** remote healthcare, surgical teleconsultation, telemedicine, telemedicine applications, telemedicine challenges

## Abstract

Telemedicine in surgical care has undergone rapid advancements in recent years, leveraging technologies such as telerobotics, artificial intelligence (AI) diagnostics, and wearable devices to facilitate remote evaluation and monitoring of patients. These innovations have improved access to care, reduced costs, and enhanced patient satisfaction. However, significant challenges remain, including technical barriers, limited tactile feedback in telesurgery, and inequities arising from digital literacy and infrastructure gaps. The rapid integration of telemedicine in surgical care necessitates a comprehensive understanding of its advancements, challenges, and implications. This review aims to consolidate existing knowledge, identify gaps, and highlight future research directions. The COVID-19 pandemic underscored telemedicine's potential, accelerating its adoption across healthcare systems worldwide. Despite these advancements, issues such as inconsistent reimbursement policies and challenges in integrating telemedicine into existing healthcare systems hinder its widespread adoption. Future research should prioritize the integration of AI, advancements in telepresence, and solutions to socioeconomic barriers to solidify telemedicine's role in global surgical care and enhance patient safety.

## Introduction and background

Telemedicine in surgery is a transformative advancement that bridges geographical distances and improves patient care. By enabling remote consultations, progress monitoring for patients and their families, and postoperative care and visits, telemedicine eliminates the need for patients to travel. It allows surgeons to collaborate effectively, providing specialized care to patients in underserved or remote places. Integrating telemedicine into surgical workflows enhances efficiency and promotes health equity by overcoming barriers to healthcare access [1].

Telemedicine refers to the use of telecommunication technology to provide healthcare services remotely. In the context of surgery, it involves using digital platforms to conduct preoperative assessments, postoperative follow-ups, and real-time multidisciplinary consultations. These applications help reduce in-person visits, benefiting patients with mobility challenges or those living in rural areas. Innovations like telerobotic surgery and augmented reality expand remote surgical capabilities, allowing precise, complex procedures from afar. This approach enhances access, streamlines processes, and improves convenience for both patients and healthcare providers [1,2].

Historically, telemedicine has evolved from early forms of telephone consultations to today’s use of advanced technologies like video conferencing, robotics, and artificial intelligence (AI) diagnostics. The integration of telemedicine into surgical care is especially significant because it offers an opportunity to address healthcare disparities by reaching underserved populations. This evolution highlights the potential of telemedicine to revolutionize healthcare delivery on a global scale.

Despite its advantages, the implementation of telemedicine in surgery faces many challenges, such as technological barriers that may not be present in all countries, patient acceptance, and regulatory hurdles. While it improves accessibility and convenience, telemedicine may not replace in-person evaluations in cases requiring physical examinations. Ongoing research and systematic reviews assess its effectiveness across surgical specialties, highlighting both benefits and limitations. As healthcare evolves with technological advancements, telemedicine shows promise for enhancing surgical care delivery and improving patient outcomes [2,3].

The rapid integration of telemedicine in surgical care necessitates a comprehensive understanding of its advancements, challenges, and implications. Technologies like telerobotics, AI diagnostics, and wearable devices have transformed patient care, yet inconsistent policies, technical limitations, and inequities in access hinder widespread adoption. This review seeks to consolidate existing knowledge, identify gaps, and highlight future research directions to guide the effective and equitable implementation of telemedicine in surgical care.

## Review

Methodology

This review was conducted using a structured approach to ensure comprehensive coverage of relevant literature on telemedicine in surgical care.

The study utilized well-established databases, including PubMed, Scopus, and Web of Science, to gather peer-reviewed articles. A robust search strategy was implemented, employing Boolean operators and keywords such as "telemedicine," "telerobotics," "AI in surgery," "wearable devices," and "remote surgical care." These terms were carefully selected to encompass the diverse applications of telemedicine within surgical practice.

The inclusion criteria for selecting studies were as follows: peer-reviewed articles published within the past 10 years to reflect contemporary advancements and challenges; studies that focused explicitly on telemedicine applications in surgical care; and research addressing technical advancements, ethical concerns, or implementation challenges.

Exclusion criteria included articles unrelated to surgical care and studies lacking original data or substantive discussion pertinent to telemedicine.

Following the initial search, abstracts, and full texts were screened to determine relevance to the review’s objectives. Selected studies underwent a thematic analysis to extract key data on technological innovations, benefits, limitations, and future directions in telemedicine for surgical care.

History of telemedicine in surgical care

Telehealth involves using technology-based platforms to deliver a range of health services, including information, prevention, monitoring, and medical care. Telemedicine, a key segment of telehealth, specifically refers to the remote practice of medicine, which can be delivered in various forms, such as doctor-to-doctor consultations or patient-to-doctor care through direct-to-consumer services [4]. 

Telemedicine emerged in the 1970s as a way to deliver healthcare remotely through technology, initially aiming to support the National Aeronautics and Space Administration (NASA) in caring for astronauts via robotic systems in different situations in their careers. The World Health Organization (WHO) defines telemedicine broadly as the remote delivery of healthcare services, utilizing information technology to gather essential data for diagnosis, treatment, and follow-up [5]. Telemedicine uses telecommunications to diagnose and treat patients in distant locations, a field that has rapidly gained traction in both medicine and surgery. Initially called "telehealth" and focused solely on medical care, it has since expanded across various disciplines, including surgery, with practices like telesurgery and tele-clinics for postoperative follow-up for evaluation of their condition. Advances in computer science and engineering now enable surgeons to enhance patient care, mentor, collaborate, and teach without geographic limitations [6].

Telesurgery began in the 1970s with systems like da Vinci® and Zeus® but is still evolving for regular use. Besides technical hurdles, telesurgery faces regulatory, billing, and liability challenges that complicate its expansion across state and national borders for its good results [7]. Advances have been made in telementoring, allowing specialist surgeons to guide local surgeons through telepresence. Despite these developments, telesurgery remains in its early stages, with major challenges around cost-effectiveness, bandwidth availability, regulations, and adoption. Another limitation is the lack of tactile feedback, which removes the crucial sense of touch from the surgeon’s hands [8].

**Figure 1:**
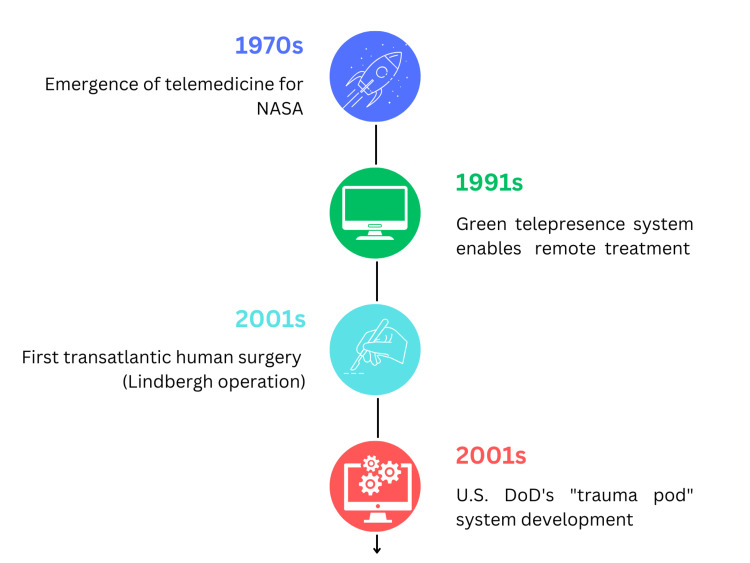
Evolution of telemedicine and telesurgery NASA: National Aeronautics and Space Administration; DoD: U.S. Department of Defense This figure has been created by the authors of this article summarizing the evolution of telemedicine and telesurgery based on references [7-8].

In 1991, the Green telepresence system enabled physicians to treat remote patients using mechatronic devices for the first time. By 2025, the U.S. Department of Defense (DoD) aims to develop the "Trauma Pod" system, allowing combat surgeons to perform critical surgeries on injured soldiers from a safe distance without travel. Launched by the Defense Advanced Research Projects Agency (DARPA) in 1994, the Trauma Pod project focuses on advancing battlefield medical care by creating autonomous and semi-autonomous mobile platforms that integrate telerobotic and robotic medical systems. From 1994 to 2003, the Institute for Research into Cancer of the Digestive System (IRCAD) in France collaborated with Computer Motion Inc. on numerous experimental procedures. Following six cholecystectomies on pigs, the first transatlantic human surgery, known as the Lindbergh operation, took place on September 7, 2001. During this laparoscopic cholecystectomy, Marescaux and his team remotely controlled the Zeus robot from New York, while the patient was located 7,000 kilometers away in Strasbourg (Figure 1) [8].

Types and models of telemedicine in surgery

Telemedicine has emerged as a transformative force in surgical care, encompassing various types and models that enhance patient access and streamline surgical intervention and follow-ups. One prominent model is preoperative and postoperative teleconsultation, which allows surgeons to conduct virtual assessments and follow-ups [9]. This model has gained traction due to its ability to minimize unnecessary hospital visits, thereby saving patients time and reducing the burden on healthcare systems. Studies indicate that telemedicine facilitates effective communication between patients and surgeons, enabling the sharing of medical information, such as diagnostic imaging and lab results, in real time. This approach not only expedites the decision-making process but also accommodates patients who may face challenges in attending in-person appointments or travels [10,11].

Another significant model is telesurgery, which utilizes robotic processes to perform surgical procedures far away. This innovative approach allows surgeons to operate on patients from a distance, enhancing access to specialized surgical care for individuals in remote locations. Telesurgery incorporates advanced technologies such as augmented reality (AR) and high-definition imaging, which improve the doctor’s visualization of anatomical structures during procedures [7]. Furthermore, the use of robotic systems helps mitigate human error and enhances surgical precision by eliminating hand tremors associated with traditional surgery. The integration of telesurgery not only broadens the geographic reach of surgical services but also promotes collaborative care among surgical teams across different facilities, thereby improving patient and operative results [12].

**Figure 2:**
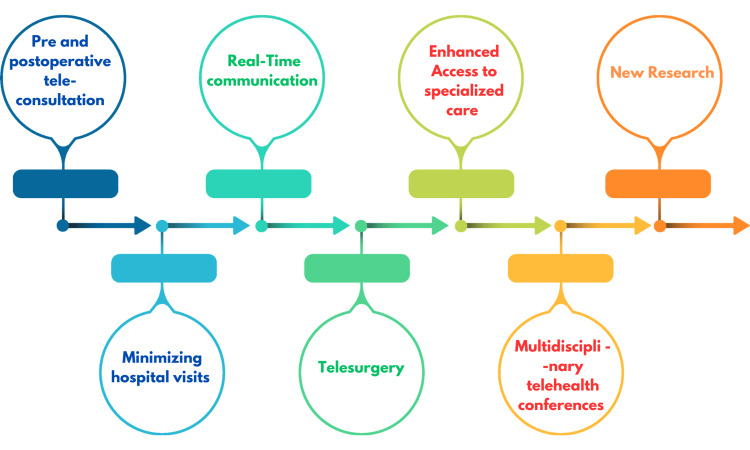
Summary of the use of telemedicine in surgical care This figure has been created by the authors of this article summarizing the evolution of telemedicine and telesurgery based on references [12,13].

These virtual meetings enable various experts, such as surgeons, radiologists, and oncologists, to collaborate on complex cases without geographical constraints. By leveraging telehealth platforms for these conferences, healthcare providers can develop comprehensive treatment plans that incorporate diverse expert opinions that help in improving outcomes. This model is particularly beneficial for managing intricate medical conditions that require coordinated efforts from multiple specialties. Overall, the diverse types and models of telemedicine in surgery not only enhance patient care but also promote efficiency within healthcare systems, demonstrating the potential for continued integration of technology in surgical practice (Figure 2) [13].

Technological infrastructure and requirements

Telemedicine in surgical care has seen rapid expansion to overcome many challenges, particularly after the COVID-19 pandemic, driven by advancements in technology and the need for enhanced virtual communication. Platforms like MyChart and Doximity are compared for their usability and integration with electronic health records (EHRs), while wearable and remote monitoring devices play a key role in improving postoperative outcomes and patient visits by linking patient data to EHRs. Additionally, telemedicine enables surgeons to telementor peers remotely without the need for visits, offering real-time observation, advice, and guidance during procedures, showcasing the potential for enhanced collaboration and improved surgical outcomes [14].

MyChart, established by Epic Systems, is broadly applied in the medical field because of its strong EHR integration. The platform improves continuity of care, particularly in postoperative monitoring, by empowering real-time access to and updating patient records by healthcare providers to decrease the efforts needed. Both patients and providers can just use MyChart because of its user-friendly interface, which was created with simplicity in mind. One drawback, though, is that MyChart's close integration with Epic's EHR system may limit its applicability in ecosystems where other EHR systems are in use [15,16].

Contrarily, Doximity is a platform that is specifically designed for healthcare professionals and offers features like text messaging and video calls to adhere to the Health Insurance Portability and Accountability Act (HIPAA). Even though it lacks an EHR component by default, it enables basic integration with several EHR systems, which makes it a more flexible option for medical practitioners who might use varying EHR systems. Although Doximity's design prioritizes quick and secure communication, which can be useful for follow-ups and surgical consultations in a real-time manner, its limitations with context to direct EHR integration may necessitate extra steps to update patient records. Doximity is therefore particularly useful in situations where secure communication is still a top concern but real-time access to EHR data is less important [12,16].

Wearable technology has become a vital component of telemedicine postoperative monitoring, offering real-time patient data that can notify medical professionals of possible issues. Vital signs like heart rate, oxygen saturation, and activity levels can be tracked by these appliances, which include biosensors and smartwatches. Electronic health records can then be linked to the gathered data, allowing medical professionals to monitor patients' recuperation from a distance. These developments in wearable technology are essential for post-surgical care because they enable continuous patient monitoring without the need for in-person visits, improving patient safety and convenience [17].

To facilitate thorough patient monitoring and prompt intervention, wearable data integration with EHRs is crucial. Although wearable technology has enormous potential for postoperative monitoring and care in many regions, the process of incorporating this data into EHR systems is still developing, as noted by Dinh-Le et al. (2019). Because it enables wearable data to be stored alongside other patient records, seamless integration is essential for providing healthcare providers with a comprehensive picture of the patient's recovery. Integration of EHR, however, frequently necessitates a large infrastructure, including secure transmission procedures and data standardization protocols, which could be problematic for smaller healthcare organizations with fewer technical resources [17].

Wearable data integration with EHRs is essential to enable comprehensive patient monitoring and timely intervention. Dinh-Le et al. (2019) point out that while wearable technology holds great promise for postoperative monitoring, the process of integrating this data into EHR systems is still in its infancy. For healthcare providers to have a complete picture of the patient's recovery, seamless integration is crucial because it allows wearable data to be stored alongside other patient records. Smaller healthcare organizations with fewer technical resources may find it difficult to integrate EHRs because they often require a large infrastructure and resources that are not always available, including secure transmission procedures and data standardization protocols [18].

Applications of telemedicine in surgical specialties

Applications of telemedicine in orthopedic, cardiovascular, and oncological surgery each present distinct needs and require specialized tools tailored to the unique challenges and procedures within each surgical field. Telemedicine in orthopedic surgery was first described in Finland 25 years ago, where doctors used to communicate and diagnose patients through video conferences [19]. Since this date, improvements in broadband internet and communication technologies have been increasing, especially for the last few years, till it has increased dramatically during the novel COVID-19 pandemic. Telemedicine has a new role during this time in decreasing viral dissemination and patient contact when doing inspection or radiological images; however, its main limitation is the inability to do a proper clinical examination. The same issue arises during rehabilitation, despite the current sensor advances in usage helping in the detection of joint movement. Therefore, a question has been raised regarding its effectiveness in orthopedics [20].

A systematic review of eight controlled randomized studies concluded there was no difference in patient satisfaction regarding communication and treatment outcomes between telemedicine applications and in-person sitting. The thing is, doctors can still show their empathy and emotions through video calls in the same way as face-to-face. The only comment was on physical examination limitations in these situations. The study's emphasis on application development would help shortly to overcome this disadvantage of telemedicine [21].

The same impact of the pandemic was noted in cardiac and vascular surgery. For instance: patient history, echocardiogram, angiogram, and image review. Tele-ECG, remotely monitoring heart rhythm through smartphones and wireless wearable sensors, is another effective example in the diagnosis of various cardiac problems during this era that helped in the management of diseased patients [22].

One retrospective study was done at the University of Texas MD Anderson Cancer Center to compare postoperative televisits and postoperative face-to-face visits within 90 days of readmission, length of hospital stay in readmission, outcome, and mortality following inpatient oncologic surgery during the COVID-19 pandemic. No differences were detected and evaluated in different views. The most common reason for readmission in the case of televisits was wound infection, and they were treated with drainage and antibiotics [23].

Challenges in adopting telemedicine in surgery

Introducing telemedicine in surgical care offers substantial benefits but faces distinct challenges that need careful handling upon implementation. Many developing regions lack comprehensive policies on telemedicine, causing uncertainty and inhibiting formal adoption [24]. Without established guidelines and specified protocols, providers and patients are exposed to legal risks, especially concerning liability in surgical consultations. Strong legal frameworks addressing data privacy, licensing, and reimbursement are necessary to build confidence in telemedicine for surgery [25,26]. Protecting patient data is also paramount, as telemedicine involves highly sensitive information, particularly in surgical cases. Without standardized protections like HIPAA in the U.S., patient data remains vulnerable to breaches, potentially affecting trust and thus slowing adoption [25].

Procedures and examinations relying on tactile or movement-based assessments cannot be replicated virtually, which impacts diagnostic precision and the quality of care. Additionally, surgeons require specific training to manage virtual assessments effectively. Without proper training, they may hesitate to adopt telemedicine practices in their daily work, further delaying its integration into surgical care and follow-ups. Addressing these skill gaps is critical to ensuring that telemedicine meets the high standards required for surgical diagnostics and patient management in different fields and subspecialties [27].

**Figure 3:**
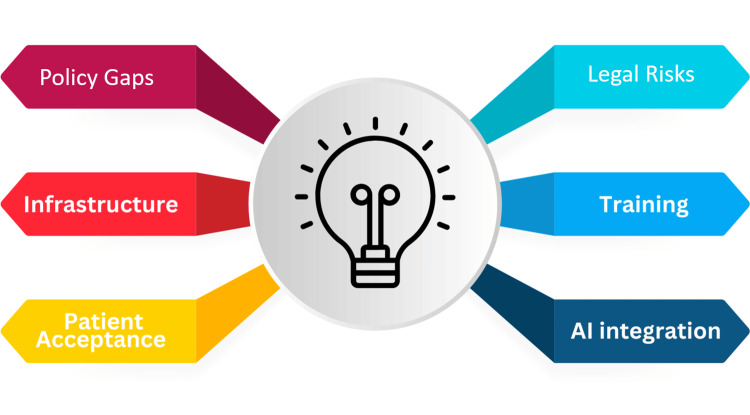
Challenges in telemedicine adoption AI: artificial intelligence This figure has been created by the authors of this article summarizing the evolution of telemedicine and telesurgery based on references [24–28].

Telemedicine in surgical care demands significant infrastructure, including high-speed internet, reliable audio-video systems, and secure data-sharing platforms, which can be arduous to implement in developing regions [24]. Patient acceptance poses another significant barrier to telemedicine adoption, as limited health and technological literacy can prevent patients from effectively engaging in virtual care and patient interaction [27,28]. This challenge is especially pronounced among lower-income groups, older adults, and rural populations, who may face additional socioeconomic obstacles. Moreover, the integration of AI into telemedicine raises trust and ethical concerns, with patients and providers needing to understand its benefits and limitations to avoid overreliance [25]. The high cost of establishing and maintaining telemedicine infrastructure, coupled with the need for continuous staff training, presents further difficulties, particularly in resource-limited settings (Figure 3) [29,30].

Telemedicine has seen rapid growth, but several significant challenges remain. Key obstacles include inadequate information infrastructure, legal and ethical issues, security concerns, and limitations in health systems and technology standards. For telemedicine to thrive globally, ongoing research is crucial to establish the effectiveness of these technologies in diagnostics and treatment. Studies have identified various barriers to telemedicine, such as poor internet bandwidth, unreliable telecommunications, and limited access to necessary technology. Issues with software compatibility and alignment with healthcare goals further complicate implementation. Additionally, unclear communication during virtual consultations and a lack of portable devices hinder usability [31].

Other studies highlight difficulties in establishing clear patient-physician interaction protocols and navigating complex financial arrangements among telemedicine partners. The privatization of healthcare and the restricted choice for patients also pose challenges, especially in developing nations. However, despite these hurdles, telemedicine presents opportunities for cost-effectiveness, improved care delivery, and enhanced international collaboration. By addressing these challenges while leveraging technological advancements, healthcare services can become more affordable and of higher quality for both providers and patients [32].

Quality of care and clinical outcome

Patient outcomes in surgical care have been significantly enhanced through the use of telemedicine. Analyzing data on surgical outcomes, readmission rates, and complication tracking reveals that telemedicine allows for effective monitoring of symptoms post-surgery. This virtual method of monitoring enables early detection of complications, which can lead to timely interventions and reduced readmission rates. Literature indicates that postoperative virtual follow-ups can significantly lower healthcare costs and readmission rates, particularly in low- and middle-income countries where traditional follow-up care is often lacking or difficult to be efficient [33,34].

Moreover, Asiri et al. (2018) explored how telemedicine impacts surgical outcomes in two key areas: patient satisfaction and perioperative care. Their systematic review found that patients engaged in virtual follow-ups often experienced outcomes comparable to, or even better than, those who attended in-person consultations and face-to-face interactions. This is particularly relevant in resource-limited settings where access to traditional healthcare may be restricted [2].

Despite the promising developments in telemedicine for surgical care, it is essential to establish clinical guidelines to ensure high-quality virtual communication. These guidelines should address remote preoperative assessments, monitoring postoperative complications, different hazards, and their management strategies. High-quality audio and video consultations are crucial for maintaining the integrity of remote evaluations [33].

As we move into a post-COVID era, there is a pressing need to review existing guidelines alongside developing new ones. A systematic review by Al Dossary et al. analyzed 164 studies on telemedicine evaluation methods in outpatient care, focusing on clinical outcomes, economic impacts, and patient satisfaction. These evaluation methods should continue to be employed as new technologies emerge [33].

Ethical and privacy considerations

Informed consent is considered the cornerstone of medical ethics, serving as both a legal requirement and a fundamental moral principle. This holds true in telemedicine, where securing a patient's informed consent is essential. The World Medical Association's (WMA) Statement on Guiding Principles for the Use of Telehealth for the Provision of Health Care emphasizes the necessity of openly providing all essential information regarding telemedicine, including the processes involved and potential limitations and liabilities. This practice ensures patient autonomy and informed decision-making. However, legal and ethical requirements for obtaining informed consent can vary depending on the region and setting in which care is provided. For example, certain regions may require additional documentation or processes to meet local legal standards. Furthermore, obtaining informed consent virtually can pose challenges not present in traditional face-to-face settings, such as the absence of non-verbal cues and visual aids. To overcome these obstacles, healthcare providers should use clear and direct language, ensure patient comprehension through interactive dialogue, and employ visual aids when necessary. Creating a supportive and accommodating environment during telemedicine sessions fosters patient involvement and preserves autonomy [33,34].

A significant challenge in telemedicine, particularly in surgical care, is ensuring equitable access for patients, as the elderly, rural communities, and individuals with low digital literacy may suffer because of this. The WMA asserts that telehealth services should be founded on values that guarantee equitable access for everyone, regardless of socioeconomic background or location. However, the technical prerequisites of telemedicine, like reliable internet access and sufficient digital literacy, can disadvantage underserved populations. These disparities are exacerbated by the digital divide, as those with fewer resources may lack the necessary technology or internet infrastructure. Without targeted efforts to enhance access, telemedicine risks widening the healthcare gap. In this context, informed consent is crucial; patients must be fully aware of the risks to their privacy and the telemedicine procedures. Vulnerable populations may require additional support, such as clearer explanations or visual aids, to understand their healthcare options and provide informed consent [35].

Innovative trends and future directions

Artificial intelligence is fast becoming a part of our daily lives, with healthcare being no different; AI has been shown to have a wide range of applications, from diagnostic tools to the review of imaging modalities. It has proven capabilities in enhancing the abilities of healthcare professionals and improving efficiency [36].

Telemedicine grew largely during the coronavirus pandemic, allowing clinicians to contact a large volume of patients where previously they may have been limited due to the speed of face-to-face contact [37].

Machine learning and deep learning are the heart of AI systems. These involve the application of algorithms for decision-making based on manually inputted data. This allows the algorithm to become “trained” for machine learning and allows it to make decisions based on the quality of the inputted data [37]. Evidence has suggested that machine learning, deep learning, and AI within telemedicine can revolutionize its practice and improve efficiency and cost-effectiveness [38].

Telemedicine holds promise in supporting surgical care in low-resource and austere settings, as demonstrated by the use of WhatsApp by the surgical team at Nasser Hospital in Gaza. Through this initiative, healthcare workers were able to leverage smartphone technology to facilitate consultations for weapon-inflicted injuries amidst direct military attacks. However, the severely constrained resources and ongoing disruptions significantly limit the effectiveness of telemedicine, underscoring the need for more robust and adaptable health infrastructure in conflict zones. The case series from Gaza exemplifies how telemedicine can offer critical support in trauma care during crises but also highlights the challenges of operating within conflict zones where resources and access are severely limited [39].

Telemedicine has allowed areas usually lacking medical aid to be given hitherto unknown levels of clinical care. Particular benefit is noted in postoperative care where patients are easily accessible and have an ease-of-access forum to gain aid if needed. Telemedicine has also allowed clinicians and specialists from all over the world to be able to communicate and share information and resources, leading to significant improvement in patient clinical care [40,41].

## Conclusions

Telemedicine in surgical care has revolutionized the delivery of healthcare by bridging geographical barriers, improving access, and enhancing efficiency. While it offers significant benefits such as cost savings, patient satisfaction, and advanced technologies like AI and telerobotics, challenges persist in technical, ethical, and infrastructural domains. The COVID-19 pandemic accelerated its adoption, demonstrating its potential in virtual consultations and follow-ups. However, limitations in tactile feedback, regulatory inconsistencies, and digital inequities remain barriers to widespread integration. Future advancements must address these challenges, focusing on equitable access, AI integration, and robust frameworks to ensure telemedicine's transformative role in global surgical care.

In addition, there is an urgent need to establish global standards for telemedicine practices in surgery. A coordinated international effort can facilitate the development of regulatory frameworks, ensuring that telemedicine is adopted safely and effectively across diverse healthcare settings. Furthermore, policies must focus on inclusive funding to improve infrastructure in underserved and low-resource areas, allowing telemedicine to reach its full potential in enhancing healthcare delivery worldwide.
